# Osteochondritis Dissecans of the Talar Dome and Navicular Bone in an Adolescent Athlete: A Prolonged Clinical Course

**DOI:** 10.7759/cureus.83495

**Published:** 2025-05-05

**Authors:** Matthew S Jorgensen, Neil Shah, Jeffrey Nadwodny, Renata Skov, George G. A Pujalte

**Affiliations:** 1 Family Medicine, University of Florida Health, Gainsville, USA; 2 Family Medicine, Mayo Clinic, Jacksonville, USA; 3 Family Medicine/Orthopedics and Sports Medicine, Mayo Clinic, Jacksonville, USA

**Keywords:** ankle and foot, athlete, navicular bone, osteochondritis dissecans, talar dome

## Abstract

Osteochondritis dissecans (OCD) of the foot and ankle is an uncommon condition in children, often associated with ankle inversion injuries. Its symptoms can mimic common ankle injuries, making it challenging to diagnose and potentially leading to delay in care. Therefore, it is crucial to further evaluate patients with persistent ankle pain despite negative radiographic imaging and no relief with standard treatment of ankle injuries. This case highlights the presentation, delayed diagnosis, and treatment of an 11-year-old male soccer player who experienced persistent left ankle and foot pain one year after sustaining an on-field injury with an unknown mechanism.

## Introduction

Osteochondritis dissecans (OCD) is a condition characterized by the separation of a segment of cartilage and underlying bone from the joint surface. Although the specific cause is unknown, it is believed that OCD lesions can be secondary to acute injury and/or repetitive trauma, especially in the adolescent population. In many OCD lesions of the ankle, the triggering event is an inversion ankle injury [1]. 

Although relatively rare in children with a prevalence of roughly 4%, OCD of the ankle and foot can lead to persistent pain and functional impairment, often mimicking other common ankle injuries (i.e., sprains and fractures) [2]. While both OCD and ankle sprains can present with pain and swelling, their causes are distinct. OCD results from a loss of blood supply to the bone and cartilage, whereas ankle sprains are due to ligament damage from sudden trauma. Additionally, OCD and stress fractures typically have a gradual onset of pain, in contrast to the sudden, sharp pain characteristic of ankle sprains [3]. Diagnosis of an OCD lesion of the ankle in a young athlete can be quite difficult, as radiographic imaging is often unremarkable. Additional diagnostic imaging should be pursued as prompt diagnosis and appropriate management are essential for optimal outcomes [2]. This case report aims to highlight the diagnostic challenges that led to a prolonged clinical course and delayed diagnosis with ultimately successful treatment of OCD of the talar dome and navicular bone in a young soccer player.

## Case presentation

**Figure 1:**
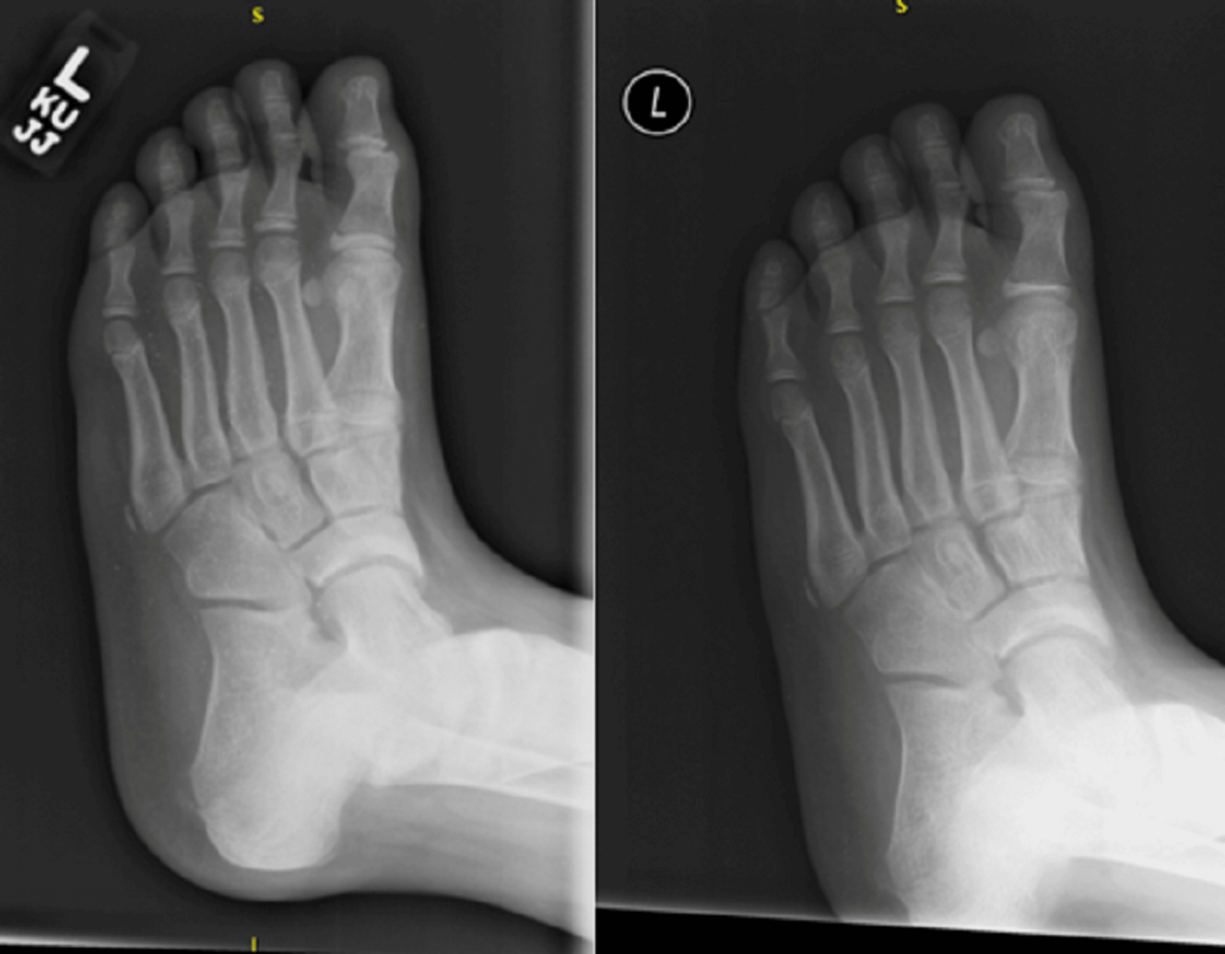
Outside hospital left ankle radiographs at one-month status post injury (A) and two-month status post injury (B) without obvious abnormalities.

**Figure 2:**
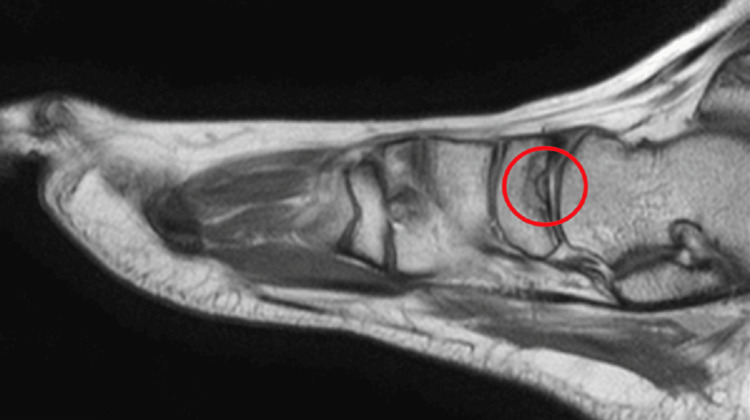
Outside hospital left ankle magnetic resonance imaging showing a subchondral navicular bone fracture with bony edema (circle).

An 11-year-old male soccer player presented to our clinic for a second opinion regarding persistent left ankle and foot pain one year after sustaining an on-field injury with an unknown mechanism. Pain was located mainly in the dorsal medial aspect of the talonavicular joint. Initial radiographs obtained at an outside institution at the time of injury were unremarkable. The athlete was initially immobilized with protected weight-bearing in a walking boot; however, his pain persisted with activity despite immobilization, causing him to refrain from playing soccer. Due to persistent pain, repeat outside radiographs were obtained at one and two months after the injury and were unremarkable (Figure 1A, 1B, respectively). A subsequent magnetic resonance image (MRI) of the ankle was obtained four months after the initial injury, which revealed a subchondral navicular bone fracture with bony edema (Figure 2). A cast was applied for four weeks with protected weight bearing. The patient was then able to gradually return to sport with shoe orthotics and an ankle stabilizing brace, with continued pain. Eight months later, his pain had not resolved without reinjury, and the athlete was unable to participate in sports. The family elected to seek a second opinion at our clinic.

On initial presentation to our clinic, the athlete demonstrated an antalgic gait favoring the left side. Heel walk was normal, but toe walk was limited due to pain. No swelling or bruising was observed, and there were no visible deformities. Point tenderness was noted over the left medial navicular region and medial talar dome, exacerbated during plantar flexion. There was a slight decrease in left ankle inversion compared to the right, but range of motion (ROM) and strength with dorsiflexion and plantar flexion were preserved. There were no mechanical limitations in ROM. The left foot and ankle were neurovascularly intact.

**Figure 3:**
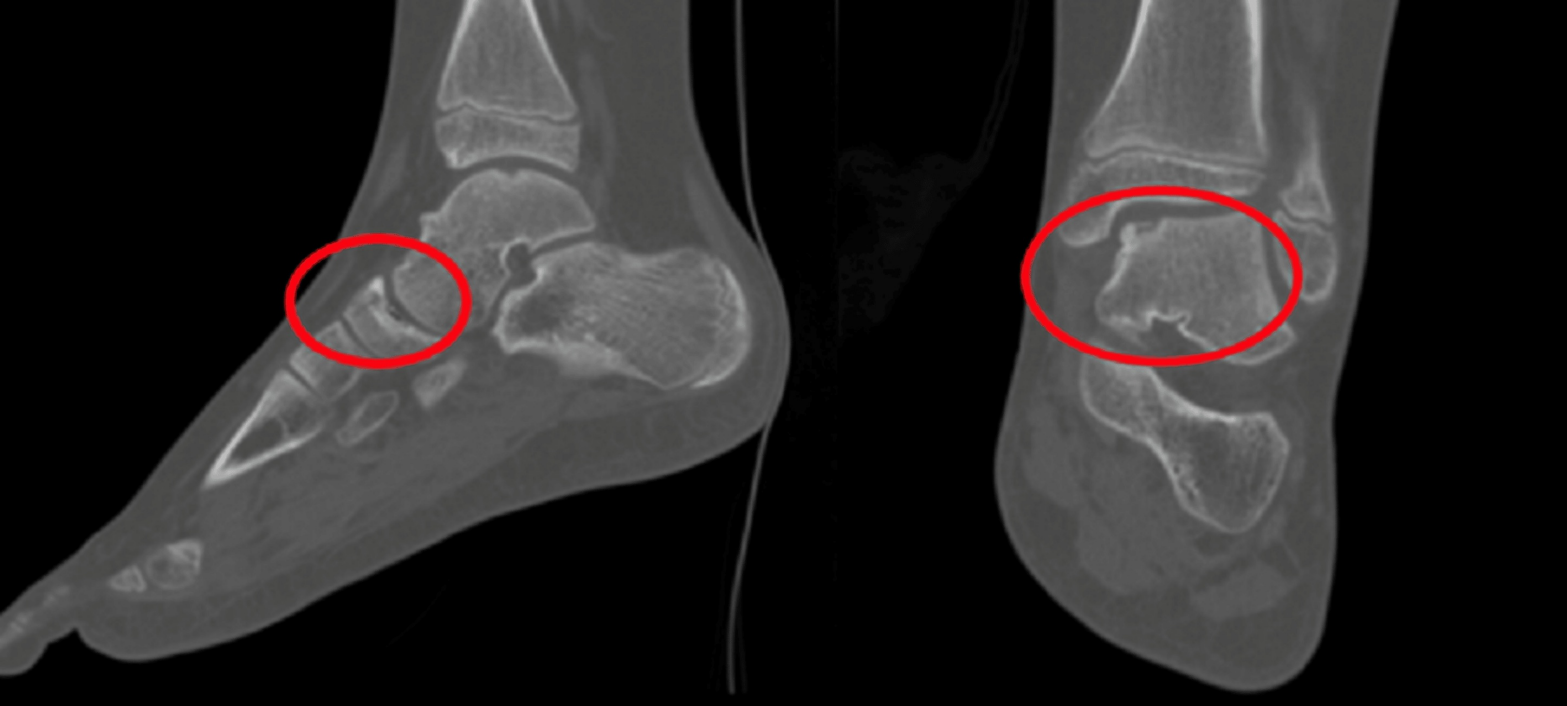
Left ankle computed tomography imaging showing osteochondral defects of the talar dome and navicular bone (Stage I-II) (circles).

Computed tomography (CT) imaging of the left foot and ankle was obtained, showing osteochondral defects of the talar dome and navicular bone (Stage I-II) (Figure 3). No unstable osteochondral fragments were noted. Both conservative and surgical options (osteochondral debridement) were discussed with the athlete and their family. Given the athlete’s lack of mechanical symptoms (i.e., clicking, catching, locking, etc.), conservative management was pursued. Treatment included four weeks of physical therapy, activity restriction, topical diclofenac gel for pain management, and custom orthotics involving ankle bracing and shoe inserts.

**Figure 4:**
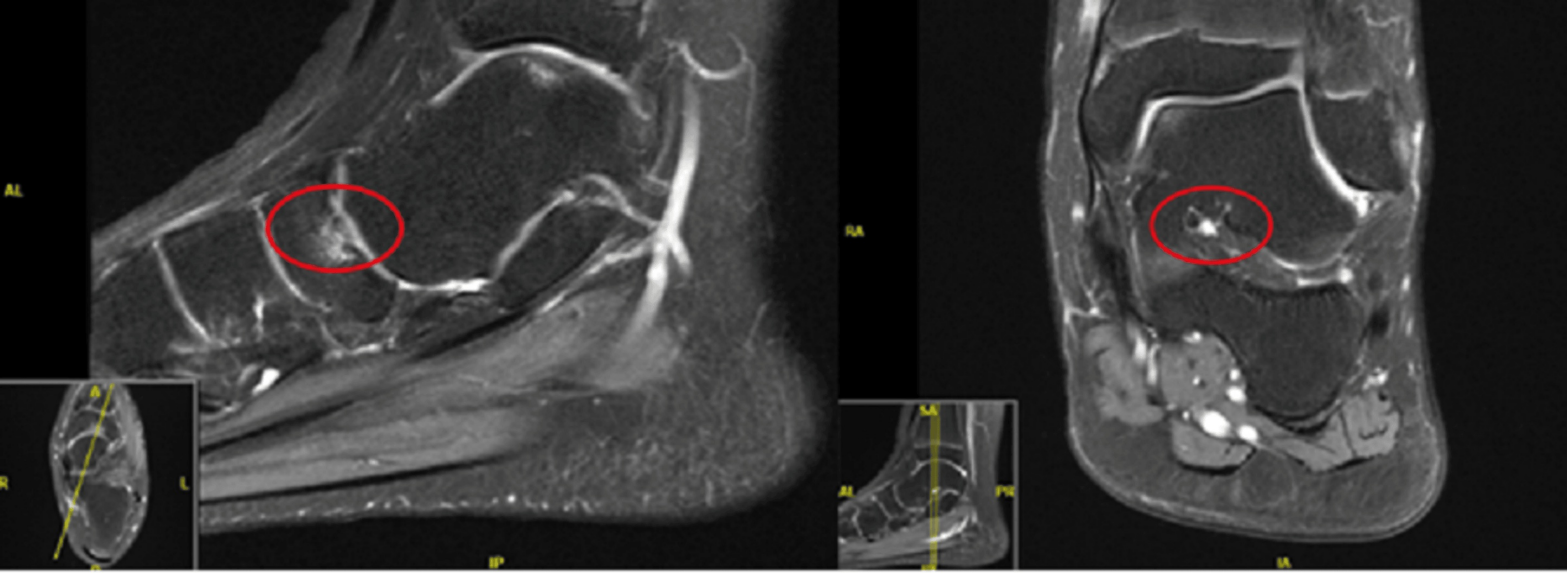
Left ankle repeat magnetic resonance imaging showing improving bony edema around the OCD lesion with no other abnormalities (red circles). OCD: osteochondritis dissecans

The athlete’s pain improved with the conservative treatment plan. He was able to return to running after four weeks of treatment. An updated MRI obtained one year after initial injury to assess interval healing showed improving bony edema around the OCD lesion with no other abnormalities (Figure 4). He was able to fully return to activity and playing soccer over one and a half years after his initial injury and has not had a recurrence of symptoms.

## Discussion

OCD is a relatively uncommon condition that primarily affects the ankle and foot, particularly in pediatric patients, where its prevalence is less than 4% [4]. The area most affected by this condition is the talar dome, often stemming from ankle inversion injuries. Making a diagnosis can be quite challenging, as initial radiographs may not show any abnormalities. In such cases, advanced imaging including CT and/or MRI is considered the gold standard for accurate diagnosis [4]. Severity of the lesion is categorized on the OCD fragment separation and ranges from Stage I (depressed fragment) to Stage IV (displaced fragment).

The primary approach to managing Stage I through III OCD lesions is initially with non-surgical conservative measures. This typically encompasses physical therapy emphasizing increased range of motion and stabilization of the affected joint, the use of non-steroidal anti-inflammatory drugs (NSAIDs) for pain relief, relative rest, activity modification, protected weight bearing, and/or immobilization [4]. Protected weight-bearing and immobilization measures include casting, splinting, controlled ankle motion (CAM) boot, crutches, and weight-bearing status and are often trialed for four to six weeks to allow for healing. Additionally, orthotics (shoe inserts, off-loaders, etc.) may be used to assist with proper joint alignment, stabilization, and offloading stress to the affected area [5]. However, in instances where these conservative measures do not improve symptoms or in patients with Stage IV lesions, surgical intervention may be required [4,6]. This is especially relevant for athletes who haven't reached skeletal maturity, as their condition could benefit from early intervention to prevent potential long-term complications, including prolonged pain, decreased sports participation, and the development of arthritis.

In the specific case discussed in this report, the athlete’s OCD was not diagnosed until roughly eight months post initial injury. The patient failed initial immobilization and activity restriction management immediately after the injury, including CAM boot (four weeks), casting (four weeks, four months post initial injury), and slow return to activity with ankle brace (five months post initial injury). After the diagnosis of OCD was made, the patient showed significant improvement through non-surgical methods including focused physical therapy, topical NSAIDS, and specially designed orthotics to offload the affected area. Over nearly two years following the initial injury, the athlete’s symptoms gradually subsided. After four weeks of conservative treatment without complete immobilization, he was able to engage in running with minimal discomfort. Approximately four months later, he fully resumed sports activities without experiencing any pain or related symptoms. The family's decision to pursue non-surgical treatment yielded successful results, where the patient’s symptoms were completely resolved with a successful return to play.

## Conclusions

While OCD of the foot and ankle is a relatively rare occurrence, it holds considerable significance, especially in pediatric athletes. Medical professionals should maintain a heightened awareness of the possibility of OCD lesions in cases of persistent ankle and foot pain that do not respond to initial treatment. A timely diagnosis, aided by CT or MRI imaging, and the implementation of appropriate management strategies, including conservative measures, can lead to favorable outcomes for these young athletes. Surgical intervention remains an option in cases where conservative management fails, leading to persistent symptoms or in cases where there are unstable OCD lesions resulting in mechanical symptoms as discussed above.
